# Economic Evaluations Alongside Efficient Study Designs Using Large Observational Datasets: the PLEASANT Trial Case Study

**DOI:** 10.1007/s40273-016-0484-y

**Published:** 2017-01-21

**Authors:** Matthew Franklin, Sarah Davis, Michelle Horspool, Wei Sun Kua, Steven Julious

**Affiliations:** 1 Health Economics and Decision Science (HEDS), ScHARR, University of Sheffield, Regent Court, 30 Regent Street, Sheffield, S1 4DA UK; 2 Design, Trials & Statistics (DTS), ScHARR, University of Sheffield, Regent Court, 30 Regent Street, Sheffield, S1 4DA UK

## Abstract

**Background:**

Large observational datasets such as Clinical Practice Research Datalink (CPRD) provide opportunities to conduct clinical studies and economic evaluations with efficient designs.

**Objectives:**

Our objectives were to report the economic evaluation methodology for a cluster randomised controlled trial (RCT) of a UK NHS-delivered public health intervention for children with asthma that was evaluated using CPRD and describe the impact of this methodology on results.

**Methods:**

CPRD identified eligible patients using predefined asthma diagnostic codes and captured 1-year pre- and post-intervention healthcare contacts (August 2012 to July 2014). Quality-adjusted life-years (QALYs) 4 months post-intervention were estimated by assigning utility values to exacerbation-related contacts; a systematic review identified these utility values because preference-based outcome measures were not collected. Bootstrapped costs were evaluated 12 months post-intervention, both with 1-year regression-based baseline adjustment (BA) and without BA (observed).

**Results:**

Of 12,179 patients recruited, 8190 (intervention 3641; control 4549) were evaluated in the primary analysis, which included patients who received the protocol-defined intervention and for whom CPRD data were available. The intervention’s per-patient incremental QALY loss was 0.00017 (bias-corrected and accelerated 95% confidence intervals [BCa 95% CI] –0.00051 to 0.00018) and cost savings were £14.74 (observed; BCa 95% CI –75.86 to 45.19) or £36.07 (BA; BCa 95% CI –77.11 to 9.67), respectively. The probability of cost savings was much higher when accounting for BA versus observed costs due to baseline cost differences between trial arms (96.3 vs. 67.3%, respectively).

**Conclusion:**

Economic evaluations using data from a large observational database without any primary data collection is feasible, informative and potentially efficient.

Clinical Trials Registration Number: ISRCTN03000938.

**Electronic supplementary material:**

The online version of this article (doi:10.1007/s40273-016-0484-y) contains supplementary material, which is available to authorized users.

## Key Points for Decision Makers

**Table:** 

Large observational datasets (such as Clinical Practice Research Datalink [CPRD]) provide opportunities to conduct clinical studies with efficient designs by utilising routinely collected resource-use data in randomised trials.
Full economic evaluations (i.e. estimation of the cost per quality-adjusted life-year [QALY]) can feasibly be conducted alongside such clinical studies by using a trial-based modelling approach to combine routinely collected data with supplementary data from the literature (such as utility values and unit costs).
This study design may be particularly suited to interventions that aim to optimise usual care and where the main clinical outcome is likely to result in a change in healthcare resource use within primary or secondary care.

## Introduction

Economic evaluations are performed alongside clinical studies to provide information to aid decision makers in regards to resource allocation. Economic evaluation requires that costs and outcomes (e.g. quality-adjusted life-years [QALYs]) are quantified, but the collection of such data can be both time consuming and costly.

Large observational datasets of routinely collected data from primary care, hospitals or wider healthcare services provide opportunities to use existing patient groups and datasets to perform studies such as randomised controlled trials (RCTs) and accompanying economic evaluations. Examples of such databases include Clinical Practice Research Datalink (CPRD) [1], ResearchOne [2] and Hospital Episode Statistics (HES) [3]. The logistical benefits of using large databases may be desirable for researchers, funding bodies looking for studies that use efficient designs and the National Health Services (NHSs) within the United Kingdom (UK) in general; such study designs have been suggested as an approach to enable patients to be entered into RCTs more quickly than traditional study designs [4]. The accurate measurement of patient-level resource-use information for the purpose of economic evaluation has historically been challenging when relying on self-reported methods [5] or raw data extracted from healthcare services [6–8]. These large observational databases provide a great deal of patient-level resource-use information, which includes data about doctors’ visits in clinic or at home, inpatient or outpatient care and prescribed drugs at the practice level—the type of data available depends on the database.

It is important to note that implementing a study within a database without any primary data collection can also generate some issues that need consideration; for example, the type and quality of data within these databases depend on the coding and recording of information at the service level. However, if a clinical condition or intervention is hypothesised to have a substantial impact on healthcare resource use within primary or secondary care, then identifying these outcomes in a large observational dataset should be feasible, and this study design could be very useful, informative and efficient for the clinical study and economic evaluation.

The PLEASANT (Preventing and Lessening Exacerbations of Asthma in School-age children Associated with a New Term) trial was a cluster RCT with a primary care-based intervention evaluated using CPRD [9]. This paper uses the PLEASANT RCT as a case study to describe the strengths and limitations of performing an economic evaluation (cost-per-QALY analysis in this case) using only data from a large observational database. The full results of the PLEASANT study are available in the health technology assessment report [10].

## Methods

### Clinical Practice Research Datalink (CPRD) and the PLEASANT Trial

CPRD is the world’s largest validated computerised database of anonymised longitudinal primary care medical records [11]. At the time of trial recruitment (January to July 2013), it included 433 contributing practices in England and Wales. Records were derived from the Vision IT General Practice software systems (although CPRD has reportedly started accepting practices using EMIS software systems [1]) and contain prescribing and coded diagnostic and clinical information as well as information on tests requested, laboratory results and referrals made at or following on from each consultation [12]. Thus, CPRD captures medical contacts, from prescription request through to out-of-hours contacts, along with contact reason. This negates the need to request this information from general practitioner (GP) practices. CPRD also captures some non-primary care contacts, such as hospital admissions; however, the comprehensiveness of this information is uncertain and relies on the relay of information between primary and secondary care [13].

A previous analysis of data from CPRD suggested there was an excess number of unscheduled contacts in children with asthma following the start of the new school term, which may in part be explained by decreased usage of medications over the summer [14]. The aim of the PLEASANT trial was to assess whether an NHS-delivered public health intervention (a letter from the GP to parents/carers of school-aged children with asthma) sent in July 2013 prior to the start of a new school term reduced the number of unscheduled medical contacts associated with asthma exacerbation after the school return in September compared with usual practice (i.e. no letter). The letter reminded parents to continue their children’s medication over the school holidays and collect medications if they were running low (the full letter is presented in the Electronic Supplementary Material [ESM] appendices, Fig. S4.1).

The study setting was primary care with practice level clustering; the recruitment processes have been published [15]. CPRD identified eligible participants based on pre-agreed asthma diagnostic codes and predefined inclusion criteria. The inclusion criteria included school-aged children (4–16 years) with a coded diagnosis of asthma, registered with a GP and receiving asthma medication during the 12-month period between March 2012 and March 2013. Patients in the intervention practices were subsequently screened by the GP to confirm inclusion. Practices randomised to intervention had to send the letter to eligible patients within the week commencing 29 July 2013; the control practices did not need to do anything. Further details about practice and patient recruitment and randomisation are provided in the ESM (Appendix S1.1).

Despite the inclusion criteria, the primary analysis population were children aged 5–16 years because of the reported difficulty associated with making an asthma diagnosis among children below this age [16, 17]; however, children aged <5 years were examined in a subgroup analysis as recommended by the Trial Steering Committee. The period of interest for exacerbations was the new school term (1 September to 31 December 2013). Resource use was assessed from 1 August 2013 to 31 July 2014 to capture any change in resource use in response to the letter intervention; data 1 year prior to intervention (1 August 2012 to 31 July 2013) were also analysed to allow adjustment for any baseline differences between trial arms.

#### Compliance with Ethical Standards

Ethical approval for the study was given by South Yorkshire Research Ethics Committee on 25 October 2012 (reference number 12/YH/04). NHS permissions to conduct the study were obtained for all the Primary Care Trusts (PCTs) in England and Health Boards in Wales. PLEASANT is Controlled Clinical Trials registered (ISRCTN03000938).

### Resource Use and Unit Costs

CPRD collect data on the number and type of medical contacts. As patients may present with multiple problems at a single contact, and contact reason is not always accurately coded at the practice level, we did not restrict our analysis to respiratory-related contacts. While not all contacts in children with asthma will be related to their asthma, we assumed that, because the practices were randomised, any difference in the number of contacts between trial arms would be related to the intervention.

An NHS perspective was taken and unit costs were assigned based on a standardised 2014/15 price year. Unit costs were assigned according to consultation type (e.g. surgery visit, hospital admission, etc.). CPRD also includes a generic entry of ‘other’ tasks, which were assumed to be unclassified administrative tasks for the purpose of applying a unit cost. All unit costs were taken from national or published sources for primary care [18–20], hospital [21] or drug [22] resource use. All unit costs are presented in the ESM (Appendixes S1.3 and S1.4, Tables S3.1–3.8), including the costing of the letter intervention (Table S3.4), which came to £1.34 per patient.

### Utility Values and the Quality-Adjusted Life-Year (QALY)

Although the UK National Institute for Health and Care Excellence (NICE) recommends trials collect preference-based patient-reported outcome measures (PB-PROMs, e.g. EuroQoL 5-Dimensions [EQ-5D]) to obtain utility values for cost-per-QALY analysis [23], PB-PROMs are not collected routinely by CPRD and were not used within the PLEASANT trial. A systematic review was used to identify exacerbation-related utility values [24]; the utility values used for this analysis [25–27] are described in Table 1. A larger utility decrement was applied for exacerbations that resulted in hospital admission (–0.2 vs. –0.1). It is worth noting that these utility decrements were based on an adult population because robust estimates in children are lacking.

**Table 1:** Health-state utility values applied in economic evaluation

Health state	Health utility value	Description of state from source study	Measurement	Source
Base-case scenario				
No exacerbation	0.96 (SD 0.07)	Average baseline utility across children (*n* = 27) aged 7–18 with GINA severity stage I–III receiving standard outpatient care in the Netherlands as part of the control arm of an RCT	EQ-5D child version (completed by parent for age <12 years). UK adult TTO valuation set	[27]
Exacerbation not requiring hospitalisation (including ED visits)	–0.10 relative to no exacerbation	Adult patients enrolled in a prospective observational study who have moderate or severe asthma (BTS rating: 4/5) at baseline and who have experienced one exacerbation requiring oral steroid treatment (without hospitalisation) in the previous 4 weeks (*n* = 22)	EQ-5D UK adult valuation set	[26]
Exacerbation requiring hospitalisation	–0.20 relative to no exacerbation	Adult patients enrolled in a prospective observational study who have moderate or severe asthma (BTS rating: 4/5) at baseline who have experienced one exacerbation requiring hospitalisation in the previous 4 weeks (*n* = 5)	EQ-5D UK adult valuation set	[26]
Sensitivity analysis				
No exacerbation	As per base case	As per base case	As per base case	[27]
Any exacerbation	–0.216 relative to no exacerbation	Patients aged >12 years (including adults) enrolled in the GOAL study who experienced an exacerbation (defined as deterioration in asthma requiring treatment with an oral corticosteroid, or an ED visit or hospitalisation)	AQLQ values mapped to EQ-5D (valuation set not stated)	[25]

*AQLQ* Asthma Quality of Life questionnaire, *BTS* British Thoracic Society, *ED* emergency department, *EQ-5D* EuroQol 5-Dimensions, *GINA* Global Initiative for Asthma, *RCT* randomized controlled trial, *SD* standard deviation, *TTO* time trade-off

CPRD contains no codes to directly determine the number, severity or duration of acute asthma exacerbations. It was therefore necessary to estimate the number of asthma exacerbations experienced from the CPRD data collected. Unscheduled contacts were assumed to represent an exacerbation (alternative exacerbation proxies and their limitations are described in the ESM [Appendix S1.2]). To define unscheduled contacts, a GP adjudication panel (consisting of three independent GPs) met, reviewed and defined the coding of the contacts recorded by CPRD as scheduled, unscheduled or not applicable (irrelevant); additional information is provided within the ESM (Appendix S1.2) and the PLEASANT website [28]. As a single exacerbation may be associated with more than one unscheduled contact, we needed to define the number of exacerbations based on the pattern of unscheduled contacts. The number of exacerbations and QALYs were calculated using a Markov assumption. We split the 4-month follow-up period into weekly cycle periods (17 × 1-week cycles and one 3-day cycle) and assumed the patient was having an exacerbation in any cycle that included an unscheduled contact of any type. Patients experiencing an exacerbation were assumed to have a utility decrement for the whole cycle period. The most severe utility decrement for a given exacerbation (i.e. hospitalised or non-hospitalised), irrespective of the number of exacerbations in a week cycle period, was applied for the whole week. QALYs were then calculated using the area under the curve (AUC) method [29].

### Statistical Analysis and Economic Evaluation

For the economic analysis, the per protocol group rather than the intention-to-treat (ITT) group was chosen to allow the economic analysis to best reflect the actual resource implications of the intervention as intended. That is, children whose parents actually received the letter (i.e. were not excluded by their GP; note, GPs would be able to exclude patients from the intervention, as appropriate, if the intervention were to be rolled out nationally) in the designated time window for the intervention to have an effect. ITT groups were assessed as part of the main clinical analysis [9].

The mean number of acute exacerbations per patient was estimated, and cost per patient was calculated by combining resource-use estimates with unit costs. Resource use is based on all ‘tasks’ recorded in CPRD. A statistically significant difference in resource use and associated costs was assessed using the *t*-test assuming unequal variance (due to the unequal sample sizes between trial arms). Statistical significance was judged at the two-sided 5% threshold, unless stated otherwise.

We assumed the intervention would have no impact on mortality and no impact on utility beyond 4 months because a previous study found excess medical contacts associated with the new school year are confined to the autumn school term (September to December; 4 months) [14]. Therefore, we expected any quality-of-life improvements from reducing exacerbations associated with the new school year to fall within the autumn term. However, it is possible the letter could have longer-term resource-use implications that were assessed over the year (e.g. being prompted to see your doctor, the doctor requesting an asthma review or picking up a prescription now may change your resource-use behaviour patterns in terms of when and how often you visit your GP practice in the future). Therefore, QALYs were calculated for 4 months post-intervention and costs were calculated for 1 year post-intervention. A sensitivity analysis that used a consistent timeframe for both costs and QALYs was conducted by analysing consequences only up until the end of December.

Accounting for baseline differences between trial arms is recommended [30–33] and should be based on patient characteristics or baseline utility values [30, 33], but, if these are not sufficiently presented, baseline costs can be used as a substitute [32]. Unit costs were attached to the resource use of the patient 1 year before the intervention to elicit 1-year baseline costs. Patient costs were adjusted by 1-year baseline costs (baseline adjusted [BA]) using bootstrapped ordinary least squares (OLS) regression models (1000 replications) with 1-year baseline costs and intervention group as covariates in the model. Nonparametric bootstrapped estimation was used for unadjusted patient costs and QALYs (1000 replications). Practice-level clustering with random effects was accounted for in the bootstrapped analysis. Unadjusted (observed) and adjusted (BA) results are reported for mean and incremental values as well as the bootstrapped standard error (bSE) and bias-corrected and accelerated confidence intervals (95% BCa CIs) [34] for all post-bootstrap estimations. For the BA mean cost estimations (not BA incremental results), the reported SEs are delta-method SEs, which are appropriate for adjusted/transformed cost approximations [35], and normal 95% CIs. The main sensitivity and subgroup analyses are described in Table 2. The point estimate incremental cost-effectiveness ratios (ICERs) were calculated as the difference in mean cost over difference in mean QALYs between the letter (Cost_L_; QALY_L_) and no letter (Cost_NL_; QALY_NL_) arms such that:

$${\text{ICER}} = \frac{{{\text{Cost}}_{\text{L}} - {\text{Cost}}_{\text{NL}} }}{{{\text{QALY}}_{\text{L}} - {\text{QALY}}_{\text{NL}} }}$$

**Table 2:** Summary of sensitivity and subgroup analysis

Model aspect varied	Base-case scenario	Sensitivity scenarios	Rationale
Unit cost for contact types defined as ‘other’	Unit cost of £0.11, assuming that ‘other’ are undefined administrative tasks	Pooled weighted unit cost of £45.58 based on the recorded resource use for all contacts and associated unit costs excluding ‘other’ tasks	Whether these ‘other’ consultation types are administrative is uncertain
Duration of exacerbation period	1 week	3 days 2 weeks	The average duration of symptoms for an exacerbation is uncertain
Utility decrement values for exacerbation	–0.1 (non-hospital) or –0.2 (hospitalisation) for exacerbation [20]	–0.216 relative to no exacerbation [19]	The utility decrement relative to no exacerbation is uncertain
Type of contacts included	All contacts regardless of whether they are respiratory related	Respiratory-related contacts	Contacts coded as respiratory related are more likely to be affected by the intervention, but a large proportion of contacts could not be coded as respiratory or non-respiratory related
QALY and cost-estimation period	QALYs estimated for 4 months and costs for 1 year post-intervention	QALYs estimated for 4 months and costs for 5 months post-intervention	To assess the shorter term (5 months) cost implications of the intervention
Age of population receiving intervention	Children aged 5–16 years	Children aged <5 years (children aged 4 years)	To assess the cost effectiveness of the intervention for children aged <5 years

*QALY* quality-adjusted life-year

The ICERs from the bootstrapped (observed and BA) analysis using 1000 replications were used to create cost-effectiveness acceptability curves (CEACs) for a range of decision makers’ willingness-to-pay (WTP) thresholds. Statistical analysis was performed using Stata version 14 [36].

## Results

### Descriptive Statistics

Of 141 practices (12,179 participants) recruited to the PLEASANT trial as of July 2013, a total of 70 practices (5917 participants) were allocated to the ‘letter’ intervention and 71 practices (6262 participants) to ‘no letter’. Of 5917 letter arm participants, 786 were excluded from the intervention by their GP and six practices (695 participants) were not eligible for the per protocol group because the letter was not sent on time or at all. Another ten letter arm practices (635 patients) and 17 no letter arm practices (1455 patients) were excluded because CPRD data were not available for the trial period, because of moving to a different GP system (i.e. not Vision). The per protocol group was used for the economic analysis, for which 8608 patients were eligible. Of these, 8190 patients (letter 3641; no letter 4549) were aged 5–16 years and used as the primary group for analysis; another 418 patients (letter 160; no letter 258) were aged <5 years and included in the subgroup analysis. A practice and patient flow CONSORT diagram is available in the ESM (Fig. S4.2).

For the primary analysis patient cohort, the mean age was 10.8 years (median 11.0 years) and 60.4% were male, both of which were consistent between trial arms (see also Table 3). The mean number of exacerbations, resource use and associated costs per patient by classified resource-use type (i.e. scheduled, unscheduled or ‘not relevant’ contacts), prescription costs and overall costs for 1-year baseline and follow-up are presented in Table 3. These results suggest that, at baseline, the letter versus no letter arm had a statistically significantly higher mean total cost of care if statistical significance is judged at a 10% threshold for descriptive purposes (£761 vs. £727, respectively;* p* = 0.069).

**Table 3:** Descriptive statistics of baseline patient characteristics, number of exacerbations (over 4 months), contacts and cost (over 12 months) pre-intervention and post-intervention per patient by trial arm

Patient characteristics	Baseline (1 September 2013)		Post-intervention	
	Letter (*N* = 3641)	No letter (*N* = 4549)	Letter (*N* = 3641)	No letter (*N* = 4549)
Age, mean (median [range])	10.8 (11 [5–16])	10.8 (11 [5–16])	NA	NA
Sex, M/F (%)	M 60.2; F 39.8	M 60.6; F 39.4	NA	NA

Exacerbations; resource-use; costs	Baseline (pre-intervention, 12 months)		Post-intervention (exacerbations, 4 months; contacts and costs, 12 months)	
Mean no. of exacerbations (SD [range]) (exacerbation period: 1 week)	NA	NA	2.50 (2.19 [0–14])	2.41 (2.19 [0–16])
Mean no. of contacts* (SD [range])				
Scheduled	2.77 (2.63 [0–29])	2.74 (2.69 [0—36])	2.60 (2.72 [0—22])	2.69 (2.85 [0—30])
Unscheduled	10.53 (8.03 [0–79])	10.44 (8.67 [0–127])	9.39 (8.32 [0–73])	9.36 (9.22 [0–101])
‘Not relevant’	4.31 (4.48 [0–52])	3.97 (4.54 [0–60])	4.17 (4.79 [0–45])	3.75 (4.73 [0–60])
Total no. contacts^a^	17.61 (12.47 [0–115])	17.14 (13.38 [0–168])	16.16 (13.30 [0–120])	15.80 (14.42 [0–163])
Mean costs (95% CI), median (range)				
Scheduled	178 (167–190), 41 (0–3871)	160 (150–169), 41 (0–5554)	169 (158–181), 27 (0–3857)	173 (163–183), 41 (0–3839)
Unscheduled	305 295–316), 208 (0–3181)	315 (305–326), 212 (0–4027)	266 (255–277), 146 (0–4661)	283 (272–294), 186 (0–8010)
‘Not relevant’	215 (202–228), 1 (0–5727)	197 (186–209), 1 (0–4111)	204 (191–217), 1 (0–6149)	205 (193–218), 1 (0–7675)
Total contact costs^b^	699 (672–725), 435 (0–8597)	672 (649–696), 408 (0–8919)	639 (612–667), 342 (0–8829)	662 (636–688), 358 (0–13,411)
Prescriptions	62 (59–66), 27 (0–1141)	55 (52–57), 20 (0–808)	55 (52–59), 20 (0–849)	49 (47–52), 16 (0–789)
Total costs^c^	761 (734–789), 498 (0–8622)	727 (703–751), 468 (0–8997)	695 (666–723), 402 (0–8921)	711 (684–738), 412 (0–13,484)
Intervention	0	0	1.34	0
Total costs and intervention cost^d^	761 (734–789), 498 (0–8622)*	727 (703–751), 468 (0–8997)*	696 (668–725), 403 (1–8922)^#^	711 (684–738), 412 (0–13,484)^#^

*T* test for unequal variance used to assess statistically significant difference in total and intervention cost between trial arms at *baseline, *p* value = 0.069 and ^#^post-intervention, *p* value = 0.460

*CI* confidence interval, *Exacs* exacerbations, *F* female, *M* male, *NA *not applicable, *No.* number, *SD* standard deviation

^a^Total number of contacts = the number of scheduled contacts *plus* the number of unscheduled contacts *plus* the number of ‘not relevant’ contacts per patient

^b^Total contact costs = the cost for scheduled contacts *plus* the cost for unscheduled contacts *plus* the cost for ‘not relevant’ contacts per patient

^c^Total costs = total contact costs *plus* the cost for the prescriptions per patient

^d^Total costs and intervention cost = Total costs *plus* the cost of the letter intervention (note, the letter intervention cost is only applied for the post-intervention period)

### Incremental Costs and QALY Results

The incremental results used to assess comparative cost effectiveness are presented in Table 4; the results by trial arm are presented in the ESM (Table S3.13), as are the patient resource use and costs by task (e.g. home visits and consultations) and trial arm (Appendix S2.1, Tables S3.9–3.12).

**Table 4:** Summary of incremental costs and QALYs, incremental cost-effectiveness ratio and cost-effectiveness results for main, adjusted, sensitivity and subgroup analysis

Analysis (1–0)^a^	Cost				QALYs				Mean ICER (£/QALY)	ICERs by CE plane quadrant (%)				Probability of CE at *λ* <WTP (%)	
	Dif. means	bSE	BCa 95% CI		Dif. means	bSE	BCa 95% CI			SE	SW	NE	NW	*λ* <£0	*λ* <£20 k
Main	–14.74	31.25	–75.86	45.19	–0.00017	0.00018	–0.00051	0.00018	88,733	14.6	52.7	2.5	30.2	67.3	63.1
BA Main	–36.07	21.10	–77.11	9.67	–0.00017	0.00018	–0.00051	0.00018	217,088	17.0	79.3	0.1	3.6	96.3	93.8
SA: ‘other’ unit cost															
Cost	14.19	36.86	–56.22	95.34	–0.00017	0.00018	–0.00051	0.00018	Dominated	11.6	26.6	5.5	56.3	38.2	34.6
BA cost	–28.53	23.64	–72.74	20.18	–0.00017	0.00018	–0.00051	0.00018	171,716	16.7	73.5	0.4	9.4	90.2	86.5
SA: duration of exacerbation															
3 days	–14.74	31.25	–75.86	45.19	–0.00005	0.00009	–0.00022	0.00012	279,489	23.5	43.8	4.3	28.4	67.3	66.0
BA 3 days	–36.07	21.10	–77.11	9.67	–0.00005	0.00009	–0.00022	0.00012	683,777	27.6	68.7	0.2	3.5	96.3	95.5
2 weeks	–14.74	31.25	–75.86	45.19	–0.00034	0.00030	–0.00093	0.00025	43,121	11.6	55.7	1.8	30.9	67.3	59.9
BA 2 weeks	–36.07	21.10	–77.11	9.67	–0.00034	0.00030	–0.00093	0.00025	105,496	13.3	83.0	0.1	3.6	96.3	90.3
SA: utility of exacerbation															
Utility	–14.74	31.25	–75.86	45.19	–0.00035	0.00038	–0.00109	0.00039	41,607	14.9	52.4	2.6	30.1	67.3	59.9
BA utility	–36.07	21.10	–77.11	9.67	–0.00035	0.00038	–0.00109	0.00039	101,793	17.4	78.9	0.1	3.6	96.3	89.8
SA: type of contacts															
Respiratory	2.41	8.65	–17.58	17.26	–0.00008	0.00005	–0.00017	0.00001	Dominated	2.0	35.5	1.9	60.6	37.5	32.5
BA respiratory	–5.06	5.98	–18.51	5.87	–0.00008	0.00005	–0.00017	0.00001	65,020	3.4	76.2	0.5	19.9	79.6	70.7
SA: cost estimation period															
5 months	3.74	14.78	–25.68	32.47	–0.00017	0.00018	–0.00051	0.00018	Dominated	11.7	29.3	5.4	53.6	41.0	34.2
BA 5 months	–6.21	10.04	–25.73	14.54	–0.00017	0.00018	–0.00051	0.00018	37,358	16.1	58.9	1.0	24.0	75.0	62.4
SG: <5 years old															
Aged <5 years	196.91	132.94	–47.60	466.99	–0.00102	0.00062	–0.00221	0.00020	Dominated	1.4	4.2	2.6	91.8	5.6	4.5
BA aged <5 years	35.69	85.30	–137.40	195.31	–0.00102	0.00062	–0.00221	0.00020	Dominated	2.8	30.6	1.2	65.4	33.4	26.3

CE plane quadrants are SE (less costly, more effective), SW (less costly, less effective), NE (more costly, more effective), NW (more costly, less effective)

*BA* baseline adjusted, *BCa 95% CI* bias-corrected and accelerated 95% confidence intervals, *bSE* bootstrapped standard error, *CE* cost effectiveness, *Dif. Means* difference in mean values between trial arms, *ICER* incremental cost-effectiveness ratio, *NE* north east, *NW* north west, *QALY* quality-adjusted life-year, *SA* sensitivity analysis, *SE* south east, *SG* subgroup analysis, *SW* south west

^a^Incremental results are the ‘letter’ group (1) minus the ‘no letter’ group (0)

For the main unadjusted analysis, the mean observed cost and QALY was £696.24 and 0.31594 QALYs for the letter group and £710.98 and 0.31611 QALYs for the no letter group. For the BA main analysis, the adjusted mean cost was £684.39 and £720.46 for the letter and no letter group, respectively (Table S3.13). The incremental mean QALY difference was –0.00017 (95% BCa CI –0.00051 to 0.00018) with a mean cost difference of –£14.74 (95% BCa CI –75.86 to 45.19) or –£36.07 (95% BCa CI –77.11 to 9.67) for the unadjusted and BA cost analysis, respectively (Table 4). Although the 95% CIs cross zero for all incremental outcomes, we can be reasonably confident that the intervention does not result in large differences in QALYs (the mean difference was equivalent to a loss of 1.5 h of perfect health) or substantial additional costs (less than the cost of one additional GP visit). The results were reasonably consistent in the sensitivity analyses using BA costs, but the subgroup analysis and some sensitivity analyses using observed costs did not estimate a mean cost saving (i.e. the intervention was less effective and more costly under these scenarios) (Table 4).

### Key Cost-Effectiveness Analysis Results

**Fig. 1:**
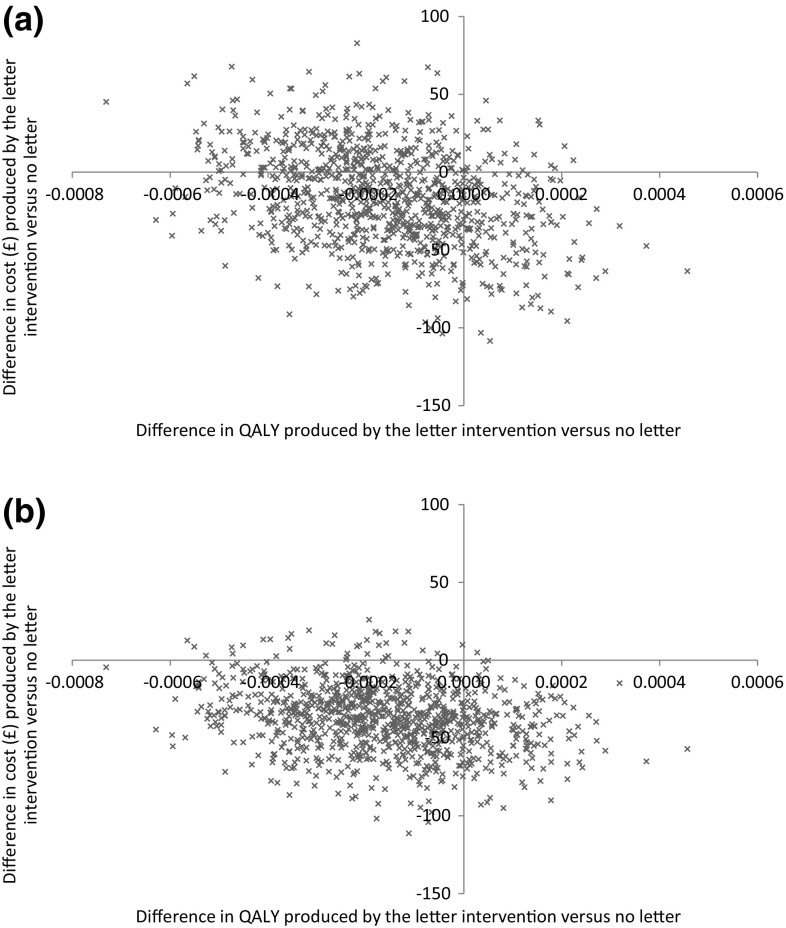
Cost effectiveness plane for the letter intervention versus no letter from the **a** main analysis and **b** baseline adjusted analysis

**Fig. 2:**
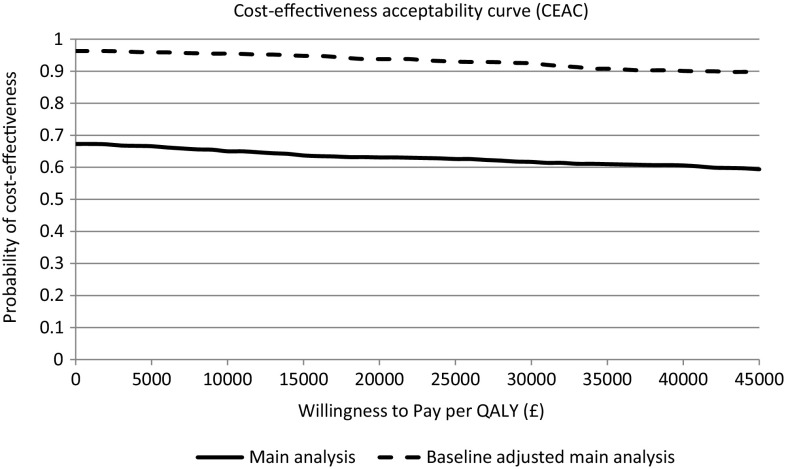
Cost effectiveness acceptability curve for the letter intervention versus no letter. Note: this graph demonstrates the probability of cost effectiveness at a range of decision-maker ceiling willingness-to-pay values for the letter intervention from the main analysis (unadjusted) and the baseline-cost adjusted main analysis

The cost-effectiveness analysis found there was some uncertainty regarding the impact of the letter intervention for both patient benefit and costs to the NHS. The differences in costs and QALYs from the bootstrapped analysis can also be visually interpreted from the cost-effectiveness planes for the unadjusted and adjusted main analysis as presented in Fig. 1a, b, respectively. Whilst the intervention was cost effective in 93.8% of samples when valuing a QALY at £20,000 in the BA analysis (Table 4; Fig. 2), it also resulted in a QALY loss within 82.9% of the bootstrapped estimates.

The very small QALY loss means the ICER is very large for all analyses. For example, for the BA main analysis, the ICER based on the mean point estimates was £217,088 per QALY, which is the ICER for the cost savings per QALY forgone, rather than the slightly more common cost per QALY gained associated with reported ICERs.

The sensitivity analyses showed that the cost-effectiveness results were sensitive to the assumptions regarding the costing of ‘other’ contacts, duration and utility decrement assigned to a period of exacerbation, types of contact included in the analysis, as well as the period of cost estimation and if the focus changed to children aged <5 years. The probability of cost effectiveness in the BA analysis for those aged 5–16 years generally remained above 62.4% at a WTP threshold (*λ*) of £20,000 per QALY and above 75.0% when focused on cost savings (rather than effectiveness; *λ* <£0) of the intervention. The probability of cost effectiveness in the BA analysis for those aged <5 years was 26.3% (*λ* <£20k) or 33.4% (*λ* <£0) (Table 4). Whilst more contacts are recorded in the letter arm (Table 3), these contacts have a lower average cost per contact, making this intervention cost saving on average in the analysis focused on those aged 5–16 years.

## Discussion

### Implications for Policy and Future Studies

To determine whether conducting economic evaluations using observational datasets is preferable to using traditional study designs, we need to consider the efficiency to researchers of using existing datasets and whether there are any trade-offs in terms of decision uncertainty.

Raw data extraction can be problematic, particularly in primary care [6]. For large studies, such as PLEASANT (with 108 practices with 12 months of data and 8190 patients), using CPRD may be considered an efficient approach, particularly given that CPRD data are relatively readily available, it is possible to plan study time horizons with expected extraction times, and a data dictionary is available to assess data availability against the needs of the study. Using data that have already been anonymised by CPRD also avoids the need for consent from individual patients, which may be necessary when directly accessing identifiable data held by practices. This was particularly efficient in this case because the intervention intended to optimise usual care, which meant it was not necessary to obtain individual consent from patients and no action was needed following randomisation in the control practices. These aspects may be desirable when commissioning or designing research studies, which could see these types of study designs being a part of research objectives from a funding body perspective.

The information provided by this study design can be described as informative because a full economic evaluation was possible using the available data. Whilst a number of assumptions were required to estimate clinical outcomes from resource-use outcomes, the probability of the intervention being cost saving remained high for the BA analysis across the sensitivity analyses. In the future, we plan to extend this work by applying expected value of perfect information (EVPI) methods [37] to undertake a more quantitative assessment of the relative value of using routine data compared with a traditional study design.

### CPRD

The PLEASANT trial was focused on a primary care-based intervention and so CPRD was ideal for this trial. Within CPRD, large amounts of primary care resource-use information are available, and CPRD also captures some resource use external to primary care, such as some hospital inpatient and emergency department data. However, this non-primary care information is not as detailed or as comprehensive as that available from other datasets such as HES. For example, CPRD records hospital admissions, but codes such as HRG-4 (resource grouping codes) are not available using CPRD but are available within HES. These codes are particularly useful for the evaluation and costing of hospital data [38], and empirical research has suggested that HES outpatient data are “reasonably valid” for research purposes [39]. However, the reliability of other aspects of HES data have been questioned [40, 41]. Also, because information about patients’ secondary care contacts must be manually entered at the practice, this information may be incomplete in primary care datasets [13]. Using linked datasets has been recommended by previous studies to best assess and evaluate the care pathway and resource use of patient groups [42, 43], but the use of linked datasets comes with its own technical and analytical challenges [44]. However, it is worth noting that a subset of English practices (reportedly 75%, representing 58% of all UK CPRD practices) have consented to participate in the CPRD linkage scheme, which includes linkage with HES data [13]. HES data were not included in this study because evidence from Cropper et al. [45] suggests that the majority of contacts for children with asthma exacerbation would occur in primary care, and we assumed that secondary care contacts would be captured in CPRD in most cases. However, we recognise this as a limitation of the study, and we may have underestimated the costs of exacerbations that resulted in secondary care contacts.

There was also an issue with practices changing IT systems away from the Vision system during the trial period, which restricted the number of patients included in the economic evaluation because of data availability. We would advise researchers designing future studies to consider the possibility of practices dropping out of the dataset when determining recruitment targets.

Herrett et al. [13] have described the representativeness and coverage of CPRD for the UK population. In terms of CPRD coverage, “The population of active patients (alive and currently registered) on 2 July 2013 was 4.4 million, representing 6.9% of the total UK population” [13]. They suggest that CPRD patients are broadly representative of the UK population in terms of age and sex [46], ethnicity [47] and body mass index (for most subgroups) [48]. CPRD practice populations have also been shown to be representative of the UK GP population, the exception being a deficit of children aged 0–4 years and an excess of patients aged ≥85 years [49]; therefore, CPRD can be considered generally representative of our primary patient group, children aged 5–16 years in the UK. There is also a question as to whether CPRD practices who know their data are being collected record activities better, are more proactive and perhaps offer better care than non-CPRD practices; however, this is probably true of any GP practice actively involved in research, so it would also affect studies using a traditional design. Furthermore, any bias is likely to be mitigated by the fact that both the quality outcomes framework (QOF) and payment by results (PbR) are informed by electronically recorded information within practices [50, 51], which provides an incentive for non-CPRD practices to also accurately record activity.

Another issue was that some practices did not implement the letter intervention, although this is an issue with trial-based evaluations in general rather than with just CPRD. This means that our analysis was restricted to the per protocol group who had data available for the trial period and sent out the letter as per the protocol. The true strength of CPRD is the logistical benefits of not having to perform primary care data collection, which is a major aspect for a more efficient study design.

### Resource Use and Cost Estimation

Whilst CPRD provides data on resource use for the costing analysis, a number of assumptions were needed to classify all the healthcare contacts as scheduled or unscheduled for the purpose of this study. We also had difficulty classifying contacts as respiratory related or not, with a large proportion (38%) remaining unclassified. The classification of resource use beyond that already coded in CPRD results in uncertainty around the estimates and causes difficulties for analysis.

We also found that a significant proportion of contacts (11.4%) were coded as consultation type ‘other’, which does not provide a clear indication of the activity involved. We therefore made an assumption regarding the type of activity that might be coded this way; however, an alternative assumption for costing ‘other’ contacts for our sensitivity analysis made some difference to the probability that the intervention was cost effective. This change in the probability of cost-effectiveness was much larger in the observed than in the BA analysis, suggesting these ‘other’ contacts were included more in the dataset for the letter than in the no letter group. This bias was controlled for in the BA analysis, but the uncertainty around the costing of these ‘other’ events is an issue when using this type of data.

The data recorded in CPRD on consultation duration and staff mix for each consultation were not considered robust enough for calculating unit costs. Therefore, we had to make assumptions using advice from our clinical experts regarding the likely staff mix and duration of contact for the purpose of applying unit costs. We also had to make assumptions regarding the likely severity of asthma exacerbations presenting in primary and secondary care.

The costing analysis for prescriptions was also problematic. A large number of different preparations are used in the management of asthma, each with a unique product code. For example, for salbutamol inhalers alone, 17 unique products were prescribed within the dataset. To keep the prescription cost analysis manageable, we estimated the cost per prescription for the ten most commonly prescribed products for each drug. This approximation is not expected to have significantly biased the cost-effectiveness analysis for this study because the absolute cost of most products prescribed in the management of asthma is low; however, such assumptions may be problematic for studies focused on medication usage.

### Utility Values and QALY Elicitation

Another limitation was that we had to infer the severity, duration and number of exacerbations experienced by patients from data on healthcare resource use to assign utility values, which required several assumptions. For example, we assumed that any week including one or more unscheduled healthcare contacts was an exacerbation week. Under this assumption, two unscheduled contacts occurring 2 days apart may count as 1 or 2 weeks of exacerbation depending on whether they fall within the same week as defined in the model. This adds uncertainty to the QALY estimates and was explored in the sensitivity analysis by varying the cycle duration from 3 days to 2 weeks. The extent to which a loss of 0.00017 QALYs (BCa 95% CI: loss of 0.00051 to a gain of 0.00018 QALYs) equivalent to a loss of 1.5 h in perfect health (BCa 95% CI: loss of 4.5 h up to a gain of 1.6 h) can be described as any tangible loss (change) in quality of life to a person is also a debatable aspect as part of this study, thus the focus has been more on the cost savings of this intervention rather than cost-effectiveness (i.e. cost per QALY).

The study’s use of routine data also meant we had to rely on published estimates for the impact of asthma exacerbations on children rather than measuring utility in the patients themselves. The systematic review did not identify any studies that directly measured exacerbation-related utility decrements in children using preference-based measures [24]. Other studies identified estimated utility decrements via mapping, either subjectively or using unpublished algorithms with insufficient details. As a result we used the ‘best available’ data from adults in the base-case analysis, but this may not accurately reflect the quality-of-life impact of exacerbations in children whose experiences of asthma and perspectives on quality-of-life may differ from those of adults. Identifying utility values from the literature is not uncommon for economic modelling, but traditional study designs can collect utility data directly from patients if needed.

### Statistical Analysis and Economic Evaluation

Previous studies have recommended that baseline resource use and cost data are collected in clinical studies to account for baseline cost differences between trial arms [31]. However, these data are not always collected, and data are often collected using retrospective self-reporting, which is subject to recall bias, which can affect the reliability of retrospectively collected resource-use information over long time horizons (such as 1 year) [5]. There are potential reasons for controlling for baseline cost differences between trial arms, particularly because of the primary care cluster design of the trial [32, 52]; for instance, (1) higher resource use and costs can be due to actual variations in care or differences in the accuracy of recording of resource use between practices, which can result in either artificial or real cost differences between trial arms; (2) a strong predictor of future resource use is past resource use, and it may be more difficult to influence the resource use habits of high resource users (frequent attenders) [53]; (3) high resource users generally have higher costs and are by nature able to have larger changes in resource use and costs than low resource users. Points (1)–(3) will influence the incremental cost difference at follow-up between trial arms if these high resource users are allocated more to one trial arm than the other because of the cluster design of the trial. For the purpose of discussion, it is unclear which of the aforementioned points may have attributed to the statistically significantly higher costs for the letter group at baseline in our case study; however, whatever the reason, this aspect was statistically controlled for in the BA analysis. Therefore, there is reason to consider that the results from the BA analysis may be a better representation of the potential economic benefit (cost savings) of the letter intervention than the unadjusted (observed) economic analysis.

## Conclusion

When designing future studies, researchers should assess the pros and cons of implementing an efficient study design within a large observational database to decide whether this design is appropriate and potentially beneficial for their trial. The main limitation with this approach is the lack of PB-PROMs on which to base utility estimates and derive QALYs. The main strengths are the logistical benefits of not having to do primary data collection, the large amounts of healthcare contact and drug data available for the purpose of analysis and the readily available resource-use information prior to intervention, which facilitates baseline adjustments. As electronic healthcare data evolve and recording quality improves, these efficient study designs may become more popular and so will the methodology for the accompanying economic evaluation.

## Electronic supplementary material

Below is the link to the electronic supplementary material.

Supplementary material 1 (DOCX 130 kb)
