# Correction to: Economic Evaluations Alongside Efficient Study Designs Using Large Observational Datasets: the PLEASANT Trial Case Study

**DOI:** 10.1007/s40273-018-00762-5

**Published:** 2019-02-16

**Authors:** Matthew Franklin, Sarah Davis, Michelle Horspool, Wei Sun Kua, Steven Julious

**Affiliations:** 1 Health Economics and Decision Science (HEDS), ScHARR, University of Sheffield, Regent Court, 30 Regent Street, Sheffield, S1 4DA UK; 2 Design, Trials & Statistics (DTS), ScHARR, University of Sheffield, Regent Court, 30 Regent Street, Sheffield, S1 4DA UK

## Correction to: PharmacoEconomics (2017) 35:561–573 10.1007/s40273-016-0484-y

The Open Access license, which previously read:

**Open Access** This article is distributed under the terms of the Creative Commons Attribution-NonCommercial 4.0International License (http://creativecommons.org/licenses/by-nc/4.0/), which permits any noncommercial use, distribution, and reproduction in any medium, provided you give appropriate credit to the original author(s) and the source, provide a link to the Creative Commons license, and indicate if changes were made.

Should read:

**Open Access** This article is distributed under the terms of the Creative Commons Attribution 4.0 International License (http://creativecommons.org/licenses/by/4.0/), which permits unrestricted use, distribution, and reproduction in any medium, provided you give appropriate credit to the original author(s) and the source, provide a link to the Creative Commons license, and indicate if changes were made.

The original article has been corrected.

