# Supplementary material for: Economic Evaluations Alongside Efficient Study Designs Using Large Observational Datasets: the PLEASANT Trial Case Study
Source: Pharmacoeconomics. 2017 Jan 21;35(5):561–73. doi: 10.1007/s40273-016-0484-y (PMC5385191; doi:10.1007/s40273-016-0484-y)
Supplement: Supplementary file 1 — Supplementary material 1 (DOCX 130 kb) [file 40273_2016_484_MOESM1_ESM.docx]

Supplementary Appendices

Economic evaluations alongside efficient study designs using large observational datasets:

the PLEASANT trial case study

Matthew Franklin^1^, Sarah Davis^1^, Michelle Horspool^1^, Wei Sun Kua^1^, and Steven Julious^1^.

^1^ School of Health and Related Research (ScHARR), University of Sheffield, Sheffield, United Kingdom.

Corresponding author’s email: matt.franklin@sheffield.ac.uk

Contents Page

Appendix S1. Supplementary Methods

S1.1. Practice recruitment and randomisation for the PLEASANT trial 2

S1.2. Methods for allocation of data to scheduled / unscheduled contacts 3

S1.3. Supplementary information on resource-use estimation 4

S1.4. Supplementary information on unit costs 5

Appendix S2. Supplementary Results

S2.1. Patient resource-use and associated cost by task for 12 months post-intervention 6

Appendix S3. Supplementary Tables

Table S3.1: Distribution of asthma severity by setting (estimated by clinical advisors) 7

Table S3.2: Costing data for unscheduled surgery visit / emergency consultation 8

Table S3.3: Costing data for primary care contacts (other than unscheduled surgery

visits / emergency consultations) 9

Table S3.4: Resource use, unit costs and total costs for letter intervention 10

Table S3.5: Average cost per prescription for drugs included in the economic analysis

(part 1 of 2) 11

Table S3.6: Average cost per prescription for drugs included in the economic analysis

(part 2 of 2) 12

Table S3.7: Unit costs for Emergency Department (ED) attendances by severity of

asthma exacerbation 13

Table S3.8: Unit costs of hospital admission 14

Table S3.9: Patient resource-use and cost by task for 12 months post-intervention

by intervention group (ordered alphabetically: A – E) 15

Table S3.10: Patient resource-use and cost by task for 12 months post-intervention

by intervention group (ordered alphabetically: F – N) 16

Table S3.11: Patient resource-use and cost by task for 12 months post-intervention

by intervention group (ordered alphabetically: O – R) 17

Table S3.12: Patient resource-use and cost by task for 12 months post-intervention

by intervention group (ordered alphabetically: S – W) 18

Table S3.13: Mean and distribution statistics by cost and QALY per patient for main,

adjusted, sensitivity analysis (SA) and subgroup analysis (SG) 19

Appendix S4. Supplementary Figures

Figure S4.1: PLEASANT intervention letter 20

Figure S4.2: CONSORT diagram of number of GP practices (N_P_) and individuals (N_I_) in

the PLEASANT study 21

Appendix S5. Supplementary References 22

Appendix S1. Supplementary Methods

S1.1. Practice recruitment and randomisation for the PLEASANT trial

Practice recruitment was carried out predominantly by CPRD. A practice recruitment pack, consisting of a detailed study information sheet and an expression of interest (EoI) form, was sent to all. This was sent by post to the preferred contact at the practice as specified in CPRD’s records for the practice. Non-responding practices were sent a reminder email, followed by a second reminder email and then final reminders by email and post. In addition to this, some practices were contacted by telephone, either by CPRD or by members of the study team at the Sheffield Clinical Trials Research Unit. Further information about practice recruitment for the PLEASANT trial has been described elsewhere [1].

Practices wishing to take part in the study, or to decline participation, returned the completed EoI form, confirming or updating as necessary the information about the practice held by CPRD. Responses were tracked by CPRD to ensure practices that had expressed interest or declined to participate were not contacted again. The EoIs were then forwarded to the study team to contact practices and complete site set up.

Primary Care Research Network also advertised and invited recruitment to the trial. Eligibility criteria for general practices were that they were using Vision IT software and agree to be signed up to CPRD if they were not already. Completed EoIs were returned to the study team for follow up and site set up.

The study team contacted interested practices to complete site set up, this was done via telephone or Skype. Once set up was complete and verbal consent obtained from the practice to participate, practice details were forwarded to the main CTRU study statistician for randomisation to one of the two trial-arms (intervention or usual care). Randomisation was at cluster (general practice) level and was stratified by size of General Practice (i.e. the “list size”) to ensure that there was an equal sample size – in terms of number of school age children with asthma – in each arm of the trial. Allocation concealment was ensured by restricting access to the CTRU statistician in regards to information about which trial-arm was represented by each arm code. The allocation was subsequently revealed to the study manager and research assistant.

The study team were unblinded throughout the study but had no access to data until after a statistical analysis plan was developed and had no influence on data capture. The GP adjudication panel did not have access to the randomisation group when reviewing the data.

Practices randomised to intervention group were sent GP packs which included the intervention letter template, which was added to practice headed paper, with procedures on confirming patient eligibility and instructions on the process and timing for delivery of the intervention via Docmail [2]. The intervention letter is presented in Figure S4.1.

Practices randomised to control group were to continue with care as usual; no other activity was required.

S1.2. Methods for the allocation of data to scheduled / unscheduled contacts and the use of unscheduled visits as a proxy for asthma exacerbations

A scheduled contact was defined as any contact that is part of the planned care for the patient, for example an asthma review; a medical review; repeat prescription or immunisation. An unscheduled contact was defined as any contact not part of their care plan that is either patient initiated or as a result of illness.

To ensure that the allocation of scheduled and unscheduled contacts was robust a GP adjudication panel consisting of 3 independent GPs attended meetings to review the data blind to treatment. The GP adjudication panel reviewed the unique terms (17% of the unique terms were reviewed which accounted for 90% of the data. During these meetings the GP adjudication panel devised the assumptions (i.e. rules) used to allocate to scheduled, unscheduled or not applicable (irrelevant) contacts. These assumptions were documented and approved by the GP adjudication panel and Trial Steering Committee (TSC; which included statisticians and professors from primary care); details provided on the PLEASANT website [3].

All types of ‘consultation’ are recorded within the data that CPRD provides; each consultation was considered a medical contact. Not all consultations are considered relevant to the study. One ‘consultation’ in the consultation table was considered one contact. All consultation data supplied are taken into account for the study not just those that are asthma related. Only consultations that happened on or after 1^st^ August 2013 were included as trial outcomes but data in the previous year were also available to allow adjustments for baseline differences.

Assumptions used to code records as scheduled, unscheduled or not applicable were based on a GP adjudication panel review of the clinical, immunisation, therapy, referral, test and consultation data.

The ‘medcode description’ from the clinical data was used first as it was felt that this table gave most description about the reason for the consultation. If contact type could not be determined by the ‘medcode description’ then clinical consultation was referenced.

Following GP adjudication panel review of the medcode descriptions and clinical consultation types, where over 90% of the data was reviewed (17% of the unique terms), clinical records were identified to be marked as scheduled (these included asthma annual review terms and other obvious types of planned appointments); unscheduled (e.g. examinations, emergency appointments); not applicable (e.g. did not attend) or unknown.

The clinical data contains more than one record per consultation and the same consultation ID can have more than one clinical contact type. For these clinical records, we assumed that unscheduled takes precedence (i.e. they are likely to have come in for an unscheduled visit but had a scheduled ‘type’ of procedure at the same time).

Consultation data marked as unknown based on the clinical data as well as consultation data that did not link to clinical data are coded based on immunisation, therapy, referral, test and consultation data. If at least one match was found in the immunisation record they were coded as scheduled. If at least one match was found with therapy (medication) data they were coded unscheduled. If they matched with the test data as part of the routine asthma review then they were coded as scheduled; if they matched with test data of peak expiratory flow rate (PEFR)/ peak flow rate (PFR) – clinical tests associated with asthma which measures speed of expiration, to monitor a person's ability to breathe out air – they were coded as unknown; otherwise a match with test data was coded as unscheduled.

Finally contacts were coded on the consultation type in the consultation table; where consultation types of follow-up / routine visits, repeat issue and medicine management were coded as scheduled; consultation types which indicate an emergency visit were coded as unscheduled; administrative type consultation types were coded as not applicable and those which were unclear, e.g. Clinic, Surgery consultation and Other were coded as unscheduled because of the likelihood that most scheduled consultation types would be clearly recorded. More details about the process of allocating consultation type codes are provided on the PLEASANT website [3].

Note, unscheduled visits were used as a proxy for exacerbations, but this required a number of assumptions as described within this section and section 2.3 of the main manuscript. An alternative proxy for exacerbations rather than unscheduled visits could have been based on prescribed asthma medications; however, after discussion with our clinical leads, medications were regarded a poorer proxy because: (i) not all asthma exacerbations result in medication being prescribed; (ii) medications prescribed for acute exacerbations can also be prescribed for chronic asthma management. Therefore, medications could either under or over-estimate the number of exacerbations due to point (i) or (ii), respectively.

S1.3. Supplementary information on resource-use estimation

For primary care contacts, the staff mix and duration of staff contact for each type of primary care contact was estimated by clinical experts (clinicians on the trial management group which included a GP and a Paediatric Respiratory Consultant). For some unscheduled surgery visits and emergency consultations, the estimates of resource use were stratified according to the severity of acute exacerbation. The proportion having moderate, severe or life-threatening exacerbation was based on clinical opinion and is shown in Table S3.1. The resource use estimates for primary care contacts are summarised in Table S3.2 and Table S3.3.

Information on the staff time required to deliver the letter intervention was based on the survey of participating practices [4] which included questions regarding the staff members involved and the duration of time required to complete the various tasks necessary to deliver the letter intervention across the eligible population within a single practice; see also the PLEASANT trial website for this report [3]. Resource use data from the survey is summarised in Table S3.4.

Data were also obtained from CPRD on the number of prescriptions for medications used in the management of chronic asthma and for medications used to treat acute exacerbations. A list of relevant drugs was prepared in consultation with clinical experts and these are shown in Table S3.5 and Table S3.6. The list of antibiotics was restricted to those commonly used to treat respiratory infections associated with asthma exacerbations in children. Although some drugs are used in the management of both chronic and acute symptoms, they have been listed in the tables under their primary use, but this has no implication for the cost-effectiveness analysis as drugs used in both indications are included in the total cost.

S1.4. Supplementary information on unit costs

For primary care contacts, the unit costs for scheduled and unscheduled patient contacts were taken from PSSRU [5] and these are summarised in Table S3.2 and Table S3.3. Department of Health reference costs [6] were used for secondary care contacts and these are summarised in Table S3.7 and Table S3.8. Drug costs were taken from the BNF for Children [7]. The average cost per prescription (see Table S5 and Table S6) was calculated by combining data on the pack size (e.g. inhaler containing 200 doses), the number of doses prescribed (e.g. 200 inhalations or 1 inhaler) and the unit cost for each item. Where more than 10 different preparations of the same drug were prescribed across the whole dataset, we have applied the average unit cost, weighted by frequency, from the 10 most commonly prescribed preparations. Unit costs for the staff time associated with delivering the intervention were based on the national costing template from the National Institute for Health Research (NIHR) Primary Care Research Network (PCRN) [8]. Unit costs for materials and postage were based on commercial costs for DocMail [2]. The total average cost per patient was estimated by combining data on resource use with the unit costs as shown in Table S3.4.

Appendix S2. Supplementary Results

S2.1. Patient resource-use and associated cost by task for 12 months post-intervention

The flow of practices and patients from point of recruitment to the point of analysis for these results are presented in a CONSORT diagram in Figure S4.1. The results presented in Table S3.9 to Table S3.12 suggest that there were some statistically significant (P < 0.05) differences in the types of tasks and associated costs that defined the patients’ resource-use over the 12 month post-intervention time period between the letter and no letter groups. For example, the no letter group had a statistically significant lower number of casualty attendances (51 [1.40%] people from the letter group had a casualty attendance compared to 6 [0.13%] from the no letter group; this resulted in a statistically significant mean higher cost of £2.84 versus £0.30 for the letter compared to the no letter group; see Table S3.9). Tasks such as “Co-op surgery consultation”, “Co-op telephone advice” and “Follow-up/routine visit” were mainly associated with the no letter group, with no recorded entry of these tasks for the letter group (see Table S3.9). Surgery consultations occurred statistically significantly more times for the no letter group, which translated into a statistically significantly higher mean cost per patient associated with these tasks of £174 for the no letter group compared to £146 for the letter group (see Table S3.12).

Tasks recorded as ‘other’ occurred statistically significantly more times for the letter group than for the no letter group, with 53.5% of the letter group having a recorded ‘other’ task compared to 45.1% for the no letter group (see also Table S3.11). For those patients that did have a recorded ‘other’ task (defined as “resource-users”), these patients had more tasks defined as ‘other’ for the letter group than the no letter group (on average, those patients that did have tasks recorded as ‘other’ had 4.1 tasks recorded as ‘other’ in the letter group compared to 3.4 for the no letter group). This resulted in a statistically significant higher mean cost of £0.24 for the letter group compared to £0.17 for the no letter group. Although this difference is very small when a unit cost of £0.11 is assigned to this ‘other’ task, larger unit costs being assigned to ‘other’ tasks could have an impact on the cost-effectiveness of the letter group as this task occurs significantly more often for this group (this aspect has been assessed as part of the sensitivity analysis described, and results of which are presented, in the main manuscript).

How these individual costs by service resulted in the overall mean costs that were used for the economic analysis are presented in Table S3.13 by trial arm for the observed and baseline adjusted analyses.

Appendix S3. Supplementary Tables

| Table S3.1: Distribution of asthma severity by setting (estimated by clinical advisors) | | |
| --- | --- | --- |
| Setting | Exacerbation type | Proportion |
| GP Surgery | Moderate asthma | 70 |
|  | Severe asthma | 25 |
|  | Life-threatening asthma | 5 |
| Emergency Department | Moderate asthma | 20 |
|  | Severe asthma | 50 |
|  | Life-threatening asthma | 30 |

| Table S3.2: Costing data for an unscheduled surgery visit/ emergency consultation | | | | | | | |
| --- | --- | --- | --- | --- | --- | --- | --- |
| Scenario for exacerbation type | Staff mix (ratio) | Duration (mins) | GP costs (£) | Nurse costs (£) | Weighted average of provider cost* (£) | Proportion of cases presented at surgery | Source |
| (i) Moderate asthma | PN or GP (20:80) | 15 | 58.50 | 13.25 | 49.45 | 0.70 | Curtis [5]^ Duration, staff mix and proportion of cases presented at surgery were estimated by clinical advisor |
| (ii) Severe asthma | | | | | | 0.25 |  |
| *Cost components:* | | | | | |  |  |
| Seeing and diagnosing patients | GP | 5 | 19.50 |  | 19.50 |  |  |
| Administration of medications | PN or GP (10:1) | 15 | 58.50 | 13.25 | 17.36 |  |  |
| Monitoring patient | PN or GP (10:1) | 5 | 19.50 | 4.42 | 5.79 |  |  |
| Total cost | | | | | 42.65 |  |  |
| (iii) Life threatening asthma  *Cost components:*  Stabilise and monitor patient until ambulance arrives | PN and GP | 5 | 19.50 | 4.42 | 23.92 | 0.05 |  |
| Unit cost of an unscheduled visit | | | | | | £46.47** |  |
| * Costs were estimated as weighted average of GP and nurse costs | | | | | | | |
| **Weighted average of unscheduled surgery costs associated with moderate, severe and life threatening asthma calculated using the proportions shown in Table S3.1. | | | | | | | |
| ^General Practitioner (GP) costs were based on £3.90 per min of patient contact, practice nurse (PN) costs were based on £53 per hour (face-to-face contact) including qualifications | | | | | | | |

| Table S3.3: Costing data for primary care contacts (other than unscheduled surgery visits / emergency consultations) | | | | | | | |
| --- | --- | --- | --- | --- | --- | --- | --- |
| Types of primary care contacts | Staff mix (ratio) | Range of duration  (mins) | Average Duration (mins) | GP costs (£)* | Nurse costs (£)** | Administrator costs (£)*** | Weighted average of provider cost (£) |
| Unscheduled clinic review | PN | 15-30 | 22.5 | - | 19.88 | - | 19.88 |
| Acute visit | GP | 10-15 | 12.5 | 48.75 | - | - | 48.75 |
| Third party consultation^ | NA | - | - | - | - | - | 212.00 |
| Unscheduled home visit | GP | 15-30 | 22.5 | 87.75 | - | - | 87.75 |
| Unscheduled phone consultation | PN:GP (10:1) | - | 5 | 19.50 | 4.42 | - | 5.79 |
| Scheduled phone consultation | PN:GP  (10:1) | - | 2 | 7.80 | 1.77 | - | 2.32 |
| Scheduled surgery consultation | PN:GP  (2:8) | 10-15 | 12.5 | 48.75 | 11.04 | - | 41.21 |
| Scheduled clinic review | PN | - | 30 | 26.50 |  | - |  |
| Medication management | PN:GP  (25:75) | - | GP: 2  PN: 5 | 7.80 | 4.42 | - | 6.95 |
| Administration | Admin | - | 0.5 | - | - | 0.11 | 0.11 |
| Results recording | Admin | - | 0.5 | - | - | 0.11 | 0.11 |
| * £3.90 per minute of patient contact for General Practitioner (GP) [5]  ** £53 per hour of face-to-face contact time for practice nurse (PN) including qualification costs [5]  *** £12.44 per hour [8]  ^ Reference cost 258: Paediatric respiratory Medicine Consultant led non-admitted face-to-face attendance, follow up, National Schedule of Reference Costs - Year 2013-14 - NHS trusts and NHS foundation trusts [6] | | | | | | | |

| Table S3.4: Resource use, unit costs and total costs for letter intervention | | | | | | | | |
| --- | --- | --- | --- | --- | --- | --- | --- | --- |
|  | | Staff | | | | | Average cost | |
|  |  | Practice Manager | Administrative Staff | GP | Practice Nurse | Research Nurse | Per practice** | Per patient** |
| *Unit cost per hour for different staff members** | | *£ 30.42* | *£ 14.15* | *£ 80.00* | *£ 0.42* | *£ 30.42* |  | |
| Resource use and average cost per task | | | | | | | | |
| *Database search* | | | | | | | | |
|  | Time per practice (minutes) | 40 | 29 | 38 | - | - |  |  |
|  | Ratio of staff involved | 38% | 46% | 17% | - | - |  |  |
|  | Weighted mean cost across staff | | | | | | £ 19.03 | £ 0.22 |
| *Check list generated by search* | | | | | | | | |
|  | Mean time per 10 patients (minutes) | 6 | 9 | 6 | 7 | 10 |  |  |
|  | Ratio of staff involved | 30% | 13% | 39% | 13% | 4% |  |  |
|  | Weighted mean cost across staff | | | | | | £ 44.31 | £ 0.51 |
| *Mail out process* | | | | | | | | |
| *Mail out by DocMail* | | | | | | | | |
|  | Time per practice (minutes) | 36 | 26 | - | - | - |  |  |
|  | Ratio of staff involved | 47% | 53% | - | - | - |  |  |
|  | Weighted mean cost across staff types | | | | | | *£ 11.92* | *£ 0.14* |
| *Mail out by other process* | | | | | | | | |
|  | Mean time per 10 patients (minutes) | 15 | 13 | - | - | - |  |  |
|  | Ratio of staff involved | 14% | 86% | - | - | - |  |  |
|  | Weighted mean cost across staff | | | | | | *£ 31.21* | *£ 0.36* |
| *DocMail cost per letter / cost of postage plus materials for other mail-out process* | | | | | | | *£ 33.69* | *£ 0.39* |
| Average across DocMail and other mail out processes (67% DocMail and 33% other) | | | | | | | £ 18.34 | £ 0.21 |
| TOTAL | | | | | | | £ 115.38 | £ 1.34 |
| * NIHR [8]  **Assuming 86 patients per practice | | | | | | | | |

| Table S3.5: Average cost per prescription for drugs included in the economic analysis (part 1 of 2) | | |
| --- | --- | --- |
| Drug class | Drug or unique combination of drugs | Average cost per prescription^  (unit cost x mean number of units) |
| Drugs used primarily in the management of chronic asthma* | | |
| Inhaled beta_2_ agonist | Salbutamol* | £2.33 |
|  | Salmeterol | £34.66 |
|  | Formoterol fumarate | £27.16 |
|  | Terbutaline | £8.64 |
| Inhaled corticosteroids | Beclometasone diproprionate | £6.71 |
|  | Budesonide | £14.29 |
|  | Fluticasone | £9.32 |
| Leukotriene receptor antagonists | Montelukast** | £4.85 |
|  | Zafirlukast | £20.80 |
| Theophylline | Modified-release oral theophylline (aminophylline /theophylline) | £5.42 |
| Cromoglicate and related therapy | Sodium cromoglycate | (Not prescribed within the dataset) |
|  | Nedocromil sodium | £34.94 |
| Combination inhalers | Beclometasone dipropionate/Formoterol fumarate dihydrate | £32.25 |
|  | Budesonide/Formoterol fumarate dihydrate | £44.27 |
|  | Fluticasone propionate/Formoterol fumarate | £30.48 |
|  | Fluticasone propionate/Salmeterol xinafoate | £32.64 |
| *Salbutamol is also used in the management of acute symptoms.  **montelukast is sometimes used in the management of acute symptoms.  ^derived by combining information on the quantity prescribed and the list price [7]. | | |

| Table S3.6: Average cost per prescription for drugs included in the economic analysis (part 2 of 2) | | |
| --- | --- | --- |
| Drug class | Drug or unique combination of drugs | Average cost per prescription^  (unit cost x mean number of units) |
| Drugs used primarily in the management of acute asthma exacerbations | | |
| Antimuscarinic bronchodilators | Ipratropium bromide | £6.43 |
| Oral corticosteroids^#^ | Oral prednisolone | £26.68 |
| Drugs used in the treatment of respiratory infections associated with asthma exacerbations | | |
| Broad-spectrum penicillins | Amoxicillin | £1.40 |
|  | Co-amoxiclav | £4.85 |
| Macrolides | Clarithromycin | £12.95 |
|  | Erythromycin | £7.61 |
| Cephalosporins | Cefaclor | £5.68 |
|  | Cefradine | (Not prescribed within the dataset) |
|  | Cefalexin | £2.22 |
| ^#^oral corticosteroids are used to manage chronic symptoms in some children.  ^derived by combining information on the quantity prescribed and the list price [7]. | | |

| Table S3.7: Unit costs for Emergency Department (ED) attendances by severity of asthma exacerbation | | | | | |
| --- | --- | --- | --- | --- | --- |
| Exacerbation type | Code | HRG | Investigations | Treatment | Unit cost* (£) |
| Moderate asthma presenting to ED | VB09Z | Emergency Medicine, Category 1 Investigation with Category 1-2 Treatment (type 1 non-admitted) | None | IV cannula, guidance advice, inhalers, oral prednisolone | 102 |
| Severe asthma presenting to ED | VB06Z | Emergency Medicine, Category 1 Investigation with Category 3-4 Treatment  (type 1 non-admitted) | Capillary blood gas | Administration of drug via spacer or nebuliser, supplemental oxygen, oral prednisolone | 128 |
| Life threatening asthma presenting to ED | VB43Z | Emergency Medicine, Category 2 Investigation with Category 4 Treatment  (type 1 non-admitted) | Capillary blood gas, chest x-ray. | Nebulisation, guidance advice, vital signs monitoring, X-Ray review, CPAP, supplemental oxygen, administration of infusion or subcutaneous drug | 224 |
| Weighted average for all Emergency Department attendances calculated using the proportions shown in Table S3.1 | | | | | 152 |
| *Sourced from the National Schedule of Reference Costs - Year 2013-14 - NHS trusts and NHS foundation trusts [6]. | | | | | |

| Table S3.8: Unit costs of hospital admission | | |
| --- | --- | --- |
| Reference cost details* | Frequency^ | Unit Costs |
| PD12C: Non-elective short stay: Paediatric Asthma or Wheezing, with CC Score 0 | 15159 | 559 |
| PD12B: Non-elective short stay: Paediatric Asthma or Wheezing, with CC Score 1-3 | 8390 | 579 |
| PD12A: Non-elective short stay: Paediatric Asthma or Wheezing, with CC Score 1-3 | 392 | 572 |
| Weighted average across all non-elective short stay admissions | | 566 |
| *Sourced from the National Schedule of Reference Costs - Year 2013-14 - NHS trusts and NHS foundation trusts [6]  ^ Frequency has been based on activity recorded for the HRG within the National Schedule of Reference Costs | | |

| Table S3.9: Patient resource-use and cost by task for 12 months post-intervention by intervention group (ordered alphabetically: A – E) | | | | | | | |
| --- | --- | --- | --- | --- | --- | --- | --- |
| Resource-use  (n = 8,190) | Letter group (n =3,641) | | | No Letter group (n =4,549) | | | Statistical Sig. Dif. |
|  | No. resource users (% study group), mean number of tasks per resource-user (SD, range) | Mean cost per patient/  £ (95% CI, median) | Mean cost per resource user /  £ (95% CI, median, range) | No. resource users (% study group), mean number of tasks per resource-user (SD, range) | Mean cost per patient/  £ (95% CI, median) | Mean cost per resource user /  £ (95% CI, median, range) | Sig. Dif. in resource-use (and costs*) between intervention groups (p-value) |
| Acute visit | 7 (0.19%),  1 (0, 1-1) | 0.09  (0.02-0.16, 0) | 48.75  (48.75-48.75, 48.75, 48.75-48.75) | 13 (0.29%),  1 (0, 1-1) | 0.14  (0.06-0.21, 0) | 48.75  (48.75-48.75, 48.75, 48.75-48.75) | 0.384 |
| Administration | 3078 (84.54%),  4.7 (4.35, 1-52) | 0.44  (0.42-0.45, 0.33) | 0.52  (0.5-0.53, 0.39, 0.11-5.72) | 3565 (78.37%),  4.25 (4.19, 1-86) | 0.37  (0.35-0.38, 0.22) | 0.47  (0.45-0.48, 0.33, 0.11-9.46) | <0.001 |
| Casualty Attendance | 51 (1.4%),  1.33 (0.77, 1-4) | 2.84 (1.95-3.73, 0) | 202.67  (169.92-235.41, 152, 152-608) | 6 (0.13%),  1.5 (0.55, 1-2) | 0.30  (0.05-0.55, 0) | 228  (140.63-315.37, 228, 152-304) | <0.001 |
| Children's Home Visit | 0 (0%),  . (., .-.) | 0  (0-0, 0) | .  (.-., ., .-.) | 1 (0.02%),  1 (., 1-1) | 0.02  (-0.02-0.06, 0) | 87.75  (.-., 87.75, 87.75-87.75) | 0.317 |
| Clinic | 804 (22.08%),  2.24 (1.73, 1-22) | 9.83  (9.03-10.62, 0) | 44.51  (42.13-46.89, 39.76, 19.88-437.36) | 851 (18.71%),  1.9 (1.44, 1-16) | 7.07 (6.51-7.63, 0) | 37.77  (35.85-39.7, 19.88, 19.88-318.08) | <0.001 |
| Co-op Surgery Consultation | 0 (0%),  . (., .-.) | 0  (0-0, 0) | .  (.-., ., .-.) | 40 (0.88%),  1.48 (0.85, 1-5) | 2.75 (1.77-3.73, 0) | 312.7  (255.28-370.12, 212, 212-1060) | <0.001 |
| Co-op Telephone advice | 0 (0%),  . (., .-.) | 0  (0-0, 0) | .  (.-., ., .-.) | 34 (0.75%),  1.41 (0.92, 1-5) | 0.06  (0.04-0.09, 0) | 8.17  (6.31-10.04, 5.79, 5.79-28.95) | <0.001 |
| Discharge details | 56 (1.54%),  2.2 (4.02, 1-31) | 0  (0-0.01, 0) | 0.24  (0.12-0.36, 0.11, 0.11-3.41) | 90 (1.98%),  1.63 (1.13, 1-6) | 0 (0-0, 0) | 0.18  (0.15-0.21, 0.11, 0.11-0.66) | 0.886 |
| Emergency Consultation | 160 (4.39%),  1.63 (1.22, 1-8) | 3.33  (2.7-3.97, 0) | 75.8  (66.94-84.67, 46.47, 46.47-371.76) | 243 (5.34%),  1.7 (1.34, 1-11) | 4.22  (3.55-4.88, 0) | 78.98  (71.11-86.85, 46.47, 46.47-511.17) | 0.058 |
| Sig. Dif: significant difference; SD: standard deviation; 95% CI: normal 95% confidence intervals  * Costs were applied to resource-use using the CPRD defined resource-use parameters (these defined types of resource-use are presented in Table S3.9 to Table S3.12 in alphabetical order); therefore, the cost is proportional to the amount of resource-use and the p-value is the same for the difference in costs and resource-use. There are instances where an unscheduled and scheduled cost was applied to the resource-use and so the p-value is not the same, in which case the p-value for the cost is provided in the parentheses if the p-value is different to that for the difference in resource-use. | | | | | | | |

| Table S3.10: Patient resource-use and cost by task for 12 months post-intervention by intervention group (ordered alphabetically: F – N) | | | | | | | |
| --- | --- | --- | --- | --- | --- | --- | --- |
| Resource-use  (n = 8,190) | Letter group (n =3,641) | | | No Letter group (n =4,549) | | | Statistical Sig. Dif. |
|  | No. resource users (% study group), mean number of tasks per resource-user (SD, range) | Mean cost per patient/  £ (95% CI, median) | Mean cost per resource user /  £ (95% CI, median, range) | No. resource users (% study group), mean number of tasks per resource-user (SD, range) | Mean cost per patient/  £ (95% CI, median) | Mean cost per resource user /  £ (95% CI, median, range) | Sig. Dif. in resource-use (and costs*) between intervention groups (p-value) |
| Follow-up/  routine visit | 0 (0%),  . (., .-.) | 0  (0-0, 0) | .  (.-., ., .-.) | 6 (0.13%),  1.5 (0.84, 1-3) | 0.08  (0.01-0.16, 0) | 63.57  (28.61-98.52, 46.47, 41.21-123.63) | 0.029  (0.027) |
| Home Visit | 10 (0.27%),  1.1 (0.32, 1-2) | 0.27  (0.09-0.44, 0) | 96.53  (76.67-116.38, 87.75, 87.75-175.5) | 12 (0.26%),  1.92 (1.73, 1-6) | 0.44  (0.11-0.78, 0) | 168.19  (71.74-264.63, 87.75, 87.75-526.5) | 0.348 |
| Hospital Admission | 10 (0.27%),  2 (1.49, 1-5) | 3.11  (0.75-5.47, 0) | 1132  (528.42-1735.58, 566, 566-2830) | 0 (0%),  . (., .-.) | 0  (0-0, 0) | .  (.-., ., .-.) | 0.01 |
| Letter from Outpatients | 168 (4.61%),  1.96 (2.08, 1-15) | 0.01  (0.01-0.01, 0) | 0.22  (0.18-0.25, 0.11, 0.11-1.65) | 122 (2.68%),  1.45 (0.98, 1-9) | 0  (0-0.01, 0) | 0.16  (0.14-0.18, 0.11, 0.11-0.99) | <0.001 |
| Mail from patient | 10 (0.27%),  2.8 (1.69, 1-5) | 0  (0-0, 0) | 0.31  (0.18-0.44, 0.28, 0.11-0.55) | 3 (0.07%),  1 (0, 1-1) | 0  (0-0, 0) | 0.11  (0.11-0.11, 0.11, 0.11-0.11) | 0.013 |
| Mail to patient | 1098 (30.16%),  1.83 (1.18, 1-7) | 0.06  (0.06-0.06, 0) | 0.2  (0.19-0.21, 0.11, 0.11-0.77) | 1362 (29.94%),  1.94 (1.41, 1-15) | 0.06  (0.06-0.07, 0) | 0.21  (0.2-0.22, 0.11, 0.11-1.65) | 0.253 |
| Medicine Management | 250 (6.87%),  1.42 (0.88, 1-6) | 0.68  (0.58-0.77, 0) | 9.87  (9.11-10.63, 6.95, 6.95-41.7) | 363 (7.98%),  2.24 (2.42, 1-20) | 1.24  (1.06-1.43, 0) | 15.58  (13.85-17.32, 6.95, 6.95-139) | <0.001 |
| Minor Injury Service | 3 (0.08%),  1 (0, 1-1) | 0.04  (-0.01-0.08, 0) | 46.47  (46.47-46.47, 46.47, 46.47-46.47) | 3 (0.07%),  1 (0, 1-1) | 0.03  (0-0.07, 0) | 46.47  (46.47-46.47, 46.47, 46.47-46.47) | 0.787 |
| NHS Direct Report | 0 (0%),  . (., .-.) | 0  (0-0, 0) | .  (.-., ., .-.) | 1 (0.02%),  1 (., 1-1) | 0  (0-0, 0) | 0.11  (.-., 0.11, 0.11-0.11) | 0.317 |
| Sig. Dif: significant difference; SD: standard deviation; 95% CI: normal 95% confidence intervals  * Costs were applied to resource-use using the CPRD defined resource-use parameters (these defined types of resource-use are presented in Table S3.9 to Table S3.12 in alphabetical order); therefore, the cost is proportional to the amount of resource-use and the p-value is the same for the difference in costs and resource-use. There are instances where an unscheduled and scheduled cost was applied to the resource-use and so the p-value is not the same, in which case the p-value for the cost is provided in the parentheses if the p-value is different to that for the difference in resource-use. | | | | | | | |

| Table S3.11: Patient resource-use and cost by task for 12 months post-intervention by intervention group (ordered alphabetically: O – R) | | | | | | | |
| --- | --- | --- | --- | --- | --- | --- | --- |
| Resource-use  (n = 8,190) | Letter group (n =3,641) | | | No Letter group (n =4,549) | | | Statistical Sig. Dif. |
|  | No. resource users (% study group), mean number of tasks per resource-user (SD, range) | Mean cost per patient/  £ (95% CI, median) | Mean cost per resource user / £ (95% CI, median, range) | No. resource users (% study group), mean number of tasks per resource-user (SD, range) | Mean cost per patient/  £ (95% CI, median) | Mean cost per resource user / £  (95% CI, median, range) | Sig. Dif. In resource-use (and costs*) between intervention groups (p-value) |
| Other | 1946 (53.45%),  4.05 (4.5, 1-44) | 0.24  (0.22-0.25, 0.11) | 0.45  (0.42-0.47, 0.33, 0.11-4.84) | 2053 (45.13%),  3.39 (3.81, 1-35) | 0.17  (0.16-0.18, 0) | 0.37  (0.35-0.39, 0.22, 0.11-3.85) | <0.001 |
| Out of hours Non Practice | 424 (11.65%),  1.68 (1.17, 1-10) | 9.07  (8.06-10.08, 0) | 77.92  (72.75-83.1, 46.47, 46.47-464.7) | 419 (9.21%),  1.65 (1.31, 1-14) | 7.08  (6.24-7.92, 0) | 76.86  (71.02-82.69, 46.47, 46.47-650.58) | 0.003 |
| Out of hours Practice | 0 (0%),  . (., .-.) | 0  (0-0, 0) | .  (.-., ., .-.) | 9 (0.2%),  1.22 (0.67, 1-3) | 0.11  (0.03-0.19, 0) | 56.8  (32.98-80.61, 46.47, 46.47-139.41) | 0.008 |
| Radiology Result | 1 (0.03%),  1 (., 1-1) | 0  (0-0, 0) | 0.11  (.-., 0.11, 0.11-0.11) | 0 (0%),  . (., .-.) | 0  (0-0, 0) | .  (.-., ., .-.) | 0.317 |
| Referral Letter | 30 (0.82%),  1.23 (0.63, 1-3) | 0  (0-0, 0) | 0.14  (0.11-0.16, 0.11, 0.11-0.33) | 5 (0.11%),  1 (0, 1-1) | 0  (0-0, 0) | 0.11  (0.11-0.11, 0.11, 0.11-0.11) | <0.001 |
| Repeat Issue | 1786 (49.05%),  4.23 (3.95, 1-30) | 0.23  (0.22-0.24, 0) | 0.47  (0.45-0.49, 0.33, 0.11-3.3) | 2293 (50.41%),  4.28 (4.51, 1-53) | 0.24  (0.23-0.25, 0.11) | 0.47  (0.45-0.49, 0.33, 0.11-5.83) | 0.307 |
| Residential Home Visit | 2 (0.05%),  1 (0, 1-1) | 0.05  (-0.02-0.12, 0) | 87.75  (87.75-87.75, 87.75, 87.75-87.75) | 4 (0.09%),  1 (0, 1-1) | 0.08  (0-0.15, 0) | 87.75  (87.75-87.75, 87.75, 87.75-87.75) | 0.574 |
| Results recording | 656 (18.02%),  2.88 (2.79, 1-28) | 0.06  (0.05-0.06, 0) | 0.32  (0.29-0.34, 0.22, 0.11-3.08) | 839 (18.44%),  3.02 (3.43, 1-38) | 0.06  (0.06-0.07, 0) | 0.33  (0.31-0.36, 0.22, 0.11-4.18) | 0.324 |
| Sig. Dif: significant difference; SD: standard deviation; 95% CI: normal 95% confidence intervals  * Costs were applied to resource-use using the CPRD defined resource-use parameters (these defined types of resource-use are presented in Table S3.9 to Table S3.12 in alphabetical order); therefore, the cost is proportional to the amount of resource-use and the p-value is the same for the difference in costs and resource-use. There are instances where an unscheduled and scheduled cost was applied to the resource-use and so the p-value is not the same, in which case the p-value for the cost is provided in the parentheses if the p-value is different to that for the difference in resource-use. | | | | | | | |

| Table S3.12: Patient resource-use and cost by task for 12 months post-intervention by intervention group (ordered alphabetically: S – W) | | | | | | | |
| --- | --- | --- | --- | --- | --- | --- | --- |
| Resource-use  (n = 8,190) | Letter group (n =3,641) | | | No Letter group (n =4,549) | | | Statistical Sig. Dif. |
|  | No. resource users (% study group), mean number of tasks per resource-user (SD, range) | Mean cost per patient/  £ (95% CI, median) | Mean cost per resource user /  £ (95% CI, median, range) | No. resource users (% study group), mean number of tasks per resource-user (SD, range) | Mean cost per patient/  £ (95% CI, median) | Mean cost per resource user /  £ (95% CI, median, range) | Sig. Dif. In resource-use (and costs*) between intervention groups (p-value) |
| Surgery consultation | 2948 (80.97%),  3.95 (3.45, 1-35) | 145.96  (140.79-151.13, 92.94) | 180.28  (174.56-186, 139.41, 41.21-1615.93) | 3762 (82.7%),  4.62 (4.61, 1-70) | 174.21  (168.15-180.27, 128.89) | 210.66  (203.89-217.43, 139.41, 41.21-3247.64) | <0.001 |
| Telephone call from a patient | 239 (6.56%),  1.42 (1.07, 1-10) | 0.51  (0.43-0.59, 0) | 7.76  (7.05-8.47, 5.79, 2.32-50.96) | 450 (9.89%),  1.7 (1.7, 1-17) | 0.92  (0.8-1.04, 0) | 9.3  (8.43-10.17, 5.79, 2.32-94.96) | <0.001 |
| Telephone call to a patient | 321 (8.82%),  1.66 (1.56, 1-14) | 0.76  (0.65-0.87, 0) | 8.62  (7.74-9.5, 5.79, 2.32-74.12) | 663 (14.57%),  2.01 (2.86, 1-52) | 1.54  (1.34-1.74, 0) | 10.56  (9.38-11.75, 5.79, 2.32-276.79) | <0.001 |
| Third Party Consultation | 2118 (58.17%),  3.73 (3.92, 1-40) | 460.04  (435.87-484.21, 212) | 790.85  (755.45-826.24, 424, 212-8480) | 2599 (57.13%),  3.79 (4.12, 1-53) | 458.72  (436.31-481.13, 212) | 802.89  (769.27-836.51, 424, 212-11236) | 0.937 |
| Triage | 359 (9.86%),  2.33 (2.25, 1-21) | 1.27  (1.09-1.45, 0) | 12.92  (11.61-14.22, 5.79, 2.32-121.59) | 640 (14.07%),  2.27 (1.92, 1-18) | 1.76  (1.59-1.93, 0) | 12.51  (11.7-13.31, 8.11, 2.32-90.34) | <0.001 |
| Twilight Visit | 1 (0.03%),  1 (., 1-1) | 0.01  (-0.01-0.04, 0) | 46.47  (.-., 46.47, 46.47-46.47) | 1 (0.02%),  1 (., 1-1) | 0.01  (-0.01-0.03, 0) | 46.47  (.-., 46.47, 46.47-46.47) | 0.876 |
| Walk-in Centre | 28 (0.77%),  1.46 (0.88, 1-5) | 0.52  (0.3-0.75, 0) | 68.05  (52.17-83.92, 46.47, 46.47-232.35) | 0 (0%),  . (., .-.) | 0  (0-0, 0) | . (.-., ., .-.) | <0.001 |
| Sig. Dif: significant difference; SD: standard deviation; 95% CI: normal 95% confidence intervals  * Costs were applied to resource-use using the CPRD defined resource-use parameters (these defined types of resource-use are presented in Table S3.9 to Table S3.12 in alphabetical order); therefore, the cost is proportional to the amount of resource-use and the p-value is the same for the difference in costs and resource-use. There are instances where an unscheduled and scheduled cost was applied to the resource-use and so the p-value is not the same, in which case the p-value for the cost is provided in the parentheses if the p-value is different to that for the difference in resource-use. | | | | | | | |

| Table S3.13: Mean and distribution statistics by cost and QALY per patient for main, adjusted, sensitivity analysis (SA) and subgroup analysis (SG) | | | | | | | | | |
| --- | --- | --- | --- | --- | --- | --- | --- | --- | --- |
| Analysis | Letter | Cost (£) | | | | QALYs | | | |
|  | (Y/ N) | Mean | Std. Err. | 95% CI* | | Mean | Std. Err. | 95% CI* | |
| Main | Y | 696.24 | 23.11 | 649.31 | 740.98 | 0.31594 | 0.00013 | 0.31567 | 0.31619 |
|  | N | 710.98 | 20.81 | 670.07 | 752.73 | 0.31611 | 0.00012 | 0.31585 | 0.31631 |
| BA Main | Y | 684.39 | 17.08 | 650.93 | 717.86 | 0.31594 | 0.00013 | 0.31567 | 0.31619 |
|  | N | 720.46 | 12.33 | 696.29 | 744.64 | 0.31611 | 0.00012 | 0.31585 | 0.31631 |
| SA: ‘other’ unit cost | | | | |  |  |  |  |  |
| Cost | Y | 794.72 | 27.19 | 747.51 | 853.65 | 0.31594 | 0.00013 | 0.31567 | 0.31619 |
|  | N | 780.53 | 24.07 | 733.49 | 826.41 | 0.31611 | 0.00012 | 0.31585 | 0.31631 |
| BA cost | Y | 770.99 | 18.74 | 734.26 | 807.72 | 0.31594 | 0.00013 | 0.31567 | 0.31619 |
|  | N | 799.52 | 13.91 | 772.26 | 826.79 | 0.31611 | 0.00012 | 0.31585 | 0.31631 |
| SA: duration of exacerbation | | | | |  |  |  |  |  |
| Three days | Y | 696.24 | 23.11 | 649.31 | 740.98 | 0.31843 | 0.00006 | 0.31830 | 0.31855 |
|  | N | 710.98 | 20.81 | 670.07 | 752.73 | 0.31848 | 0.00006 | 0.31835 | 0.31858 |
| BA three days | Y | 684.39 | 17.08 | 650.93 | 717.86 | 0.31843 | 0.00006 | 0.31830 | 0.31855 |
|  | N | 720.46 | 12.33 | 696.29 | 744.64 | 0.31848 | 0.00006 | 0.31835 | 0.31858 |
| Two weeks | Y | 696.24 | 23.11 | 649.31 | 740.98 | 0.31236 | 0.00022 | 0.31190 | 0.31276 |
|  | N | 710.98 | 20.81 | 670.07 | 752.73 | 0.31270 | 0.00020 | 0.31226 | 0.31304 |
| BA two weeks | Y | 684.39 | 17.08 | 650.93 | 717.86 | 0.31236 | 0.00022 | 0.31190 | 0.31276 |
|  | N | 720.46 | 12.33 | 696.29 | 744.64 | 0.31270 | 0.00020 | 0.31226 | 0.31304 |
| SA: utility of exacerbation | | | | |  |  |  |  |  |
| Utility | Y | 696.24 | 23.11 | 649.31 | 740.98 | 0.31048 | 0.00028 | 0.30989 | 0.31100 |
|  | N | 710.98 | 20.81 | 670.07 | 752.73 | 0.31083 | 0.00025 | 0.31028 | 0.31126 |
| BA utility | Y | 684.39 | 17.08 | 650.93 | 717.86 | 0.31048 | 0.00028 | 0.30989 | 0.31100 |
|  | N | 720.46 | 12.33 | 696.29 | 744.64 | 0.31083 | 0.00025 | 0.31028 | 0.31126 |
| SA: type of contacts | | | | |  |  |  |  |  |
| Respiratory | Y | 123.17 | 4.98 | 114.10 | 133.39 | 0.31999 | 0.00004 | 0.31992 | 0.32006 |
|  | N | 120.76 | 6.87 | 108.90 | 136.88 | 0.32007 | 0.00003 | 0.32001 | 0.32013 |
| BA Respiratory | Y | 119.02 | 3.17 | 112.80 | 125.23 | 0.31999 | 0.00004 | 0.31992 | 0.32006 |
|  | N | 124.08 | 3.17 | 114.42 | 133.74 | 0.32007 | 0.00003 | 0.32001 | 0.32013 |
| SA: cost estimation period | | |  |  |  |  |  |  |  |
| Five months | Y | 322.70 | 9.64 | 303.47 | 341.75 | 0.31594 | 0.00013 | 0.31567 | 0.31619 |
|  | N | 318.96 | 11.15 | 298.17 | 340.84 | 0.31611 | 0.00012 | 0.31585 | 0.31631 |
| BA five month | Y | 317.17 | 7.03 | 303.40 | 330.94 | 0.31594 | 0.00013 | 0.31567 | 0.31619 |
|  | N | 323.38 | 7.13 | 309.40 | 337.35 | 0.31611 | 0.00012 | 0.31585 | 0.31631 |
| SG: under 5 years old | |  |  |  |  |  |  |  |  |
| Under 5’s | Y | 1006.21 | 120.73 | 798.09 | 1289.15 | 0.31397 | 0.00049 | 0.31285 | 0.31485 |
|  | N | 809.30 | 53.23 | 722.15 | 932.29 | 0.31500 | 0.00038 | 0.31407 | 0.31561 |
| BA under 5’s | Y | 906.71 | 67.80 | 773.82 | 1039.59 | 0.31397 | 0.00049 | 0.31285 | 0.31485 |
|  | N | 871.01 | 50.85 | 771.35 | 970.67 | 0.31500 | 0.00038 | 0.31407 | 0.31561 |
| QALY: quality adjusted life year; BA: baseline adjusted; SE: standard error; 95% CI: 95% confidence intervals; SA: sensitivity analysis; SG: subgroup analysis.  * All unadjusted SE are bias-corrected and 95% CI are bias-corrected and accelerated based on estimates post-bootstrap; however, adjusted SE are delta-method SEs and 95% CI are normal 95% CIs. | | | | | | | | | |

Appendix S4. Supplementary Figures

Figure S4.1: The PLEASANT letter template

Note: all parts of the letter marked within “< >” are to be edited as appropriate for the patient of interest

**<GP letterhead>**

< Address line 1>

< Address line 2>

< Address line 3>

< Address line 4>

<Insert Date>

Dear Parent

**Please read this important letter regarding your child’s asthma**

It is really important that your child continues to take their asthma medication during the summer holidays. Returning to school is a time when asthma can get worse and make children and young people with asthma poorly. This may be due to contact with infections at the start of the new school year.

To reduce the chances of getting poorly when they return to school, your child should continue to take their asthma medication as prescribed by their GP or practice nurse. If your child has stopped taking their medication over the summer holidays it is important to start it again as soon as possible. If they are short of medication, or you are not sure of the proper dose, please get in touch with the practice.

Yours sincerely

<Name of Doctor>

**Practice recruitment**

No. practices recruited: N_P_ = 141

No. of patients within practices: N_I_ = 12,179

**Randomised: ‘Letter’**

No. practices (patients) randomised to the ‘letter’ trial-arm: N_P_ = 70 (N_I_ = 5,917)

**Randomised: ‘No Letter’**

No. practices (patients) randomised to the ‘no letter’ trial-arm: N_P_ = 71 (N_I_ = 6,262)

**Patients excluded by GP**

No. patients excluded by GP from receiving the letter: N_I_ = 786

**Practices not adhering to protocol**

No. of practices (patients in these practices) who did not adhere to the protocol defined intervention: N_P_ = 6 (N_I_ = 695)

**Per Protocol intervention group**

No. practices (patients) in the Per Protocol group: N_P_ = 64 (N_I_ = 4,436)

**CPRD data available for analysis**

No. practices (patients) for whom CPRD data available: N_P_ = 54 (N_I_ = 3,801)

- No. patients in **primary analysis group** (aged 5 to 16 years old):

N_I_ = 3,641

- No. patients in analysis **sub-group** (aged 4 years old): N_I_ = 160

**CPRD data NOT available for analysis**

No. practices (patients) for whom CPRD data not available for analysis: N_P_ = 10 (N_I_ = 635)

**CPRD data available for analysis**

No. practices (patients) for whom CPRD data available: N_P_ = 54 (N_I_ = 4,807)

- No. patients in **primary analysis group** (aged 5 to 16 years old):

N_I_ = 4,549

- No. patients in analysis **sub-group** (aged 4 years old): N_I_ = 258

**CPRD data NOT available for analysis**

No. practices (patients) for whom CPRD data not available for analysis: N_P_ = 17 (N_I_ = 1,455)

**Key**

N_P =_ number of practices

N_I =_ number of patients

Figure S4.2: CONSORT diagram of number of GP practices (N_P_) and individuals (N_I_) in the PLEASANT study

Note: The consort diagram shows the number of practices (N_P_) and individuals (N_I_) removed from the economic analysis due to reasons such as “GP Exclusion”, “Practices not adhering to protocol”, or “CPRD data not available for analysis” which resulted in a total of 8,190 patients (‘letter’, 3,641; ‘no letter’, 4,549) available for the primary health economic analysis; another 418 patients (‘letter’, 160; ‘no letter’, 258) were classified as the ‘under 5’s’ subgroup (i.e. aged 4 years old based on the inclusion criteria).

Appendix S5. Supplementary References

1. Horspool MJ, Julious SA, Mooney C, May R, Sully B, Smithson WH. Preventing and Lessening Exacerbations of Asthma in School-aged children Associated with a New Term (PLEASANT): Recruiting Primary Care Research Sites–the PLEASANT experience. NPJ primary care respiratory medicine. 2015;25:15066.

2. DocMail. DocMail online price list. 2015. <http://www.docmail.co.uk/>.

3. PLEASANT trial. PLEASANT study website. The University of Sheffield. 2015. <https://www.shef.ac.uk/scharr/sections/dts/ctru/pleasant>.

4. Hatfield I, Julious S, Davis S, Horspool M, Norman P, Mooney C. An assessment of the resources used by General Practices in the intervention arm of the PLEASANT study in sending out the intervention. 2015.

5. Curtis L. Unit costs of health and social care. Canterbury: University of Kent. 2014.

6. Department of Health. National schedule of reference costs. Department of Health (DoH). 2014.

7. Joint Formularly Committee. BNF for Children August. 2015.

8. NIHR. Clinical Research Network Industry Costing Template. 2015.
